# Vitamin D, Diet and Musculoskeletal Health

**DOI:** 10.3390/nu15132902

**Published:** 2023-06-27

**Authors:** Deborah Agostini, Sabrina Donati Zeppa

**Affiliations:** Department of Biomolecular Sciences, University of Urbino Carlo Bo, 61029 Urbino, Italy

Vitamin D is a fat-soluble steroid hormone, acting through genomic and non-genomic mechanisms, obtainable via two main sources: diet and exposure to ultraviolet B rays. It is essential for the health of the musculoskeletal system because it improves calcium absorption, bone mineralization, and facilitates the maintenance of muscular performance. 

From fetal life to old age, adequate levels of vitamin D are necessary to maintain whole-body homeostasis and health [1], while skeletal muscle problems, as well as autoimmune and cardiovascular pathologies, diabetes, cancer, and other conditions, are known to be increased by vitamin D deficiency [2]. The vitamin D receptor (VDR), which is expressed at varying levels in different tissues, determines how much of an impact vitamin D has. However, in recent years, substantial effects have also been observed in cells with low levels of receptor expression, such as those in skeletal muscle [3].

Numerous human investigations have connected vitamin D insufficiency, reduced muscle performance, and a greater prevalence of sarcopenia [4]. Worldwide, it is estimated that one billion individuals lack a sufficient amount of vitamin D [5]. Even while integration with vitamin D is beneficial, particularly in the elderly, there is still no agreement on the recommended daily intake of vitamin D.

This Special Issue of *Nutrients*, titled “Vitamin D, Diet and Musculoskeletal Health”, includes two reviews, eleven original research articles, and one communication regarding the complex relationship between vitamin D and musculoskeletal health, with an emphasis on other variables such as nutrition, aging, illnesses, gut microbiota, and exercise.

For healthy bone growth and preservation, vitamin D is crucial, and the vitamin D activation pathway has been elucidated. The 1,25 dihydroxyvitamin D3 [1,25(OH)2D3], in particular, promotes calcium and phosphate absorption, which in turn improves bone mineralization, preventing and counteracting bone loss and fracture through a number of putative mechanisms. Even if children of a short height were shown to have low vitamin D levels, there is no conclusive proof that vitamin D affects children’s growth in the normal population. Kuraoka et al. [6] investigated the connection between vitamin D deficit and increase in height in 3624 participants, considering also the relationship between blood vitamin D levels and sun exposure. They discovered that children of all statures experienced a 0.6 cm per year reduction in height growth due to a confirmed vitamin D deficiency (10 ng/mL) and that kids with vitamin D deficiencies were less active outside, particularly in the winter. Vitamin D deficiency can also influence malocclusion development, affecting craniofacial bone growth [7]. The effect of vitamin D on the frequency of a certain fracture may vary since there are several types of fractures that happen at particular ages and under unique conditions. For this reason, Erdmann et al. [8] looked at how vitamin D can reduce the incidence of juvenile fractures, stress fractures, hip fractures, and vertebral fractures. Donati et al. demonstrates that 25 hydroxyvitamin D3 [25(OH)D3], like the physiologically active 1,25(OH)2D3, may trigger quick, non-genomic mechanisms, raising intracellular Ca^2+^ levels in mesenchymal stem cells derived from human adipose tissue in a test tube, leading to a better understanding of vitamin D3’s non-genomic effects and its function in the endocrine system [9]. The association between polymorphisms in vitamin-D-pathway-related genes, vitamin D levels, muscle mass, and function is the subject of a systematic review aimed at collating evidence on this topic [10]. The collected information could be used to choose candidate SNPs for further research, to develop individualized tactics for identifying people at risk of vitamin D insufficiency, and ultimately for assessing a possible response to vitamin D treatment.

Several studies collected in this Special Issue analyze the effect of vitamin D deficiency or supplementation on skeletal muscle health and functionality. Vitamin D insufficiency was linked to low handgrip strength, being at risk of malnutrition or malnourished, or having been diagnosed with type 2 diabetes mellitus, in elderly Mexican women living in communities [11]. Interestingly a study evaluating the vitamin D metabolism in patients affected by type 1 diabetes mellitus (T1DM) shows that the therapeutic response to cholecalciferol in patients with appropriately regulated T1DM 25(OH)D3 levels is comparable to that of healthy people [12], suggesting that supplementation, when necessary, can be useful also in the diabetic population. In rats with glucocorticoids (dexamethasone)-induced muscle atrophy, serum 25(OH)D3 concentrations were shown to be lower, and vitamin D supplementation prevented muscle loss, particularly in the soleus muscle [13].

The interaction between vitamin D and physical exercise and the effect on muscle and general health, both in physiological and pathological conditions, were investigated. The effectiveness of vitamin D in addition to aerobic interval training was shown in a single-blinded randomized controlled clinical trial in obese women vulnerable to myalgia (because of their much lower vitamin D concentration) [14]. In a double-blinded, randomized controlled trial, vitamin D administration decreased the risk of falls and increased postural stability in individuals undergoing rehabilitation four weeks after anterior cervical interbody fusion [15]. Another study compared the effects of high-intensity interval training and Nordic walking, with regard to the impact of the metabolites of vitamin D in elderly people [16]. The results showed that Nordic walking reduced the myostatin concentration more effectively than HIIT in these subjects.

The effect of vitamin D supplementation in kynurenine metabolism in healthy runners after an ultramarathon has been investigated by Mieszkowski et al. [17]. The majority of free tryptophan in humans is converted to kynurenines affecting the immunological system, the central nervous system, and the bioenergetics of skeletal muscle. After the run, the control group levels of kynurenic, xanthurenic, quinolinic, and picolinic acids considerably rose, but this effect was blunted by vitamin D supplementation; furthermore, supplementation promotes a decline in the blood levels of tryptophan, tyrosine, and phenylalanine just after the run, demonstrating an influence on tryptophan metabolism modifications.

The positive effects on energy metabolism, muscle mass, and strength in vitamin-D-deficient individuals are well known, while data on vitamin-D-sufficient individuals are lacking. An analysis on resting metabolic rate, body composition, and strength in vitamin-D-sufficient physically active young adults supplemented for 12 weeks showed no extra physiological benefits, achieving supraphysiological blood total 25(OH)D concentrations [18]. This supports the idea that supplementation only makes sense if there is a deficiency, and intervention protocols aimed at improving muscle health must necessarily consider the subject’s starting conditions.

According to the principle of individualized strategy, the last review of this Special Issue suggests a personalized integrated strategy to counteract sarcopenia and maintain the health of skeletal muscles, taking into account the role of exercise, gut microbiota health, and supplementation with antioxidants, polyunsaturated fatty acids, kefir, probiotics, prebiotics, proteins, and short-chain fatty acids as a potential aid [19].

In conclusion, this Special Issue presents new and interesting research by several scientists working in the field and gathers evidence on the role of vitamin D on musculoskeletal health. Interactions with other factors involved, such as individual polymorphisms, age, exercise, gut microbiota, etc., are also taken into consideration. Far from being a complete discussion on this fascinating and complex topic, it certainly represents an interesting starting point for further investigations.
